# Detection and elimination of pulse train instabilities in broadband fibre lasers using dispersion scan

**DOI:** 10.1038/s41598-020-64109-x

**Published:** 2020-04-29

**Authors:** Benjamín Alonso, Salvador Torres-Peiró, Rosa Romero, Paulo T. Guerreiro, Azahara Almagro-Ruiz, Héctor Muñoz-Marco, Pere Pérez-Millán, Helder Crespo

**Affiliations:** 1 Sphere Ultrafast Photonics, S.A., R. do Campo Alegre 1021, Edifício FC6, 4169-007 Porto, Portugal; 2 Grupo de Investigación en Aplicaciones del Láser y Fotónica, Departamento de Física Aplicada, University of Salamanca, E-37008 Salamanca, Spain; 3 FYLA LASER SL, Ronda Guglielmo Marconi 12, 46980 Paterna, Valencia Spain; 4 IFIMUP and Departamento de Física e Astronomia, Faculdade de Ciências, Universidade do Porto, R. do Campo Alegre 687, 4169-007 Porto, Portugal

**Keywords:** Fibre lasers, Ultrafast lasers, Supercontinuum generation, Optical metrology

## Abstract

We use self-calibrating dispersion scan to experimentally detect and quantify the presence of pulse train instabilities in ultrashort laser pulse trains. We numerically test our approach against two different types of pulse instability, namely second-order phase fluctuations and random phase instability, where the introduction of an adequate metric enables univocally quantifying the amount of instability. The approach is experimentally demonstrated with a supercontinuum fibre laser, where we observe and identify pulse train instabilities due to nonlinear propagation effects under anomalous dispersion conditions in the photonic crystal fibre used for spectral broadening. By replacing the latter with an all-normal dispersion fibre, we effectively correct the pulse train instability and increase the bandwidth of the generated coherent spectrum. This is further confirmed by temporal compression and measurement of the output pulses down to 15 fs using dispersion scan.

## Introduction

Fibre lasers are unique light sources that have an increasing number of applications in industry^1^, nonlinear^2^ and multiphoton^3^ microscopy, micro-processing^4^, generation of optical vortices^5^, vector beams^6^, high power laser development^7,8^, and imaging^9^, among many others. Important efforts have been devoted to generating ultrashort pulses from these lasers^10–16^. In particular, a very active research field is supercontinuum generation in fibre lasers^17–20^.

However, it is also well known that the supercontinuum generation process can be associated to pulse train instabilities^21–24^, which have been studied theoretically^25,26^ and experimentally with different approaches, e.g. based on interferometry^27^ and spectral-temporal analysis^23^. Regarding the characterization of stable ultrashort pulses, different techniques have been developed^28^. Over the last few years, the problem of the temporal measurement of unstable pulse trains has relied on using temporal characterization techniques such as autocorrelation, SPIDER (spectral phase interferometry for electric field reconstruction) or FROG (frequency-resolved optical gating)^29,30^. In autocorrelation, the pulse train instability is reflected in a narrow spike at the centre of the signal, which can mislead to a wrong pulse duration measurement. In interferometric measurements, such as SPIDER, previous works reported that the instability results in a reduction of contrast that cannot be experimentally identified^29^. In the case of FROG, the 2D trace is sensitive to the pulse train instability, which is associated to a higher error in the convergence^29^, very recently having been shown a specific analysis to identify the average pulse and coherent artifact contributions^30^. The effect of spectral amplitude and phase instabilities has been studied theoretically using the MIIPS (multiphoton intrapulse interference phase scan) technique and experimentally applied to a titanium-sapphire oscillator and amplifier^31,32^.

The dispersion scan (d-scan) technique^33^ enables simultaneous measurement and compression of ultrashort pulses down to single-cycle (1.04-cycle) durations^34^ and has recently been used in the theoretical study of instabilities^35,36^. D-scan is based on measuring the spectrum of a nonlinear signal (such as second-harmonic generation) produced by a pulse as a function of (usually known) dispersion applied to the pulse. The resulting two-dimensional trace (the d-scan trace) enables retrieving the spectral phase of the pulse using a numerical algorithm. Previous works report, as expected, that pulse train instabilities lead to a spreading of the d-scan trace along the dispersion axis^31,36^. This effect is due to the fact that, in the presence of instabilities, the imparted dispersion for which the spectral phase is compensated for the different wavelengths of the input pulse is also inheriting that instability, therefore the nonlinear signal is redistributed along the dispersion axis.

Here we present a d-scan-based method to experimentally assess the presence of pulse train instabilities and apply it to the measurement and optimization of supercontinuum fibre lasers. This method is based on the recently introduced self-calibrating d-scan (SC d-scan)^37^, which enables measuring a pulse with an arbitrary (i.e., unknown) compressor, since the latter’s nominal dispersion is also retrieved by the d-scan algorithm. The details of the SC d-scan technique are provided in the Methods section. Based on the retrieval of the applied dispersion, we can define a metric that effectively accounts for the instabilities. The resulting temporal characterization is therefore self-diagnosed against the pulse instabilities.

## Results and Discussion

### Theoretical study with second-order dispersion and random phase instabilities

**Figure 1:**
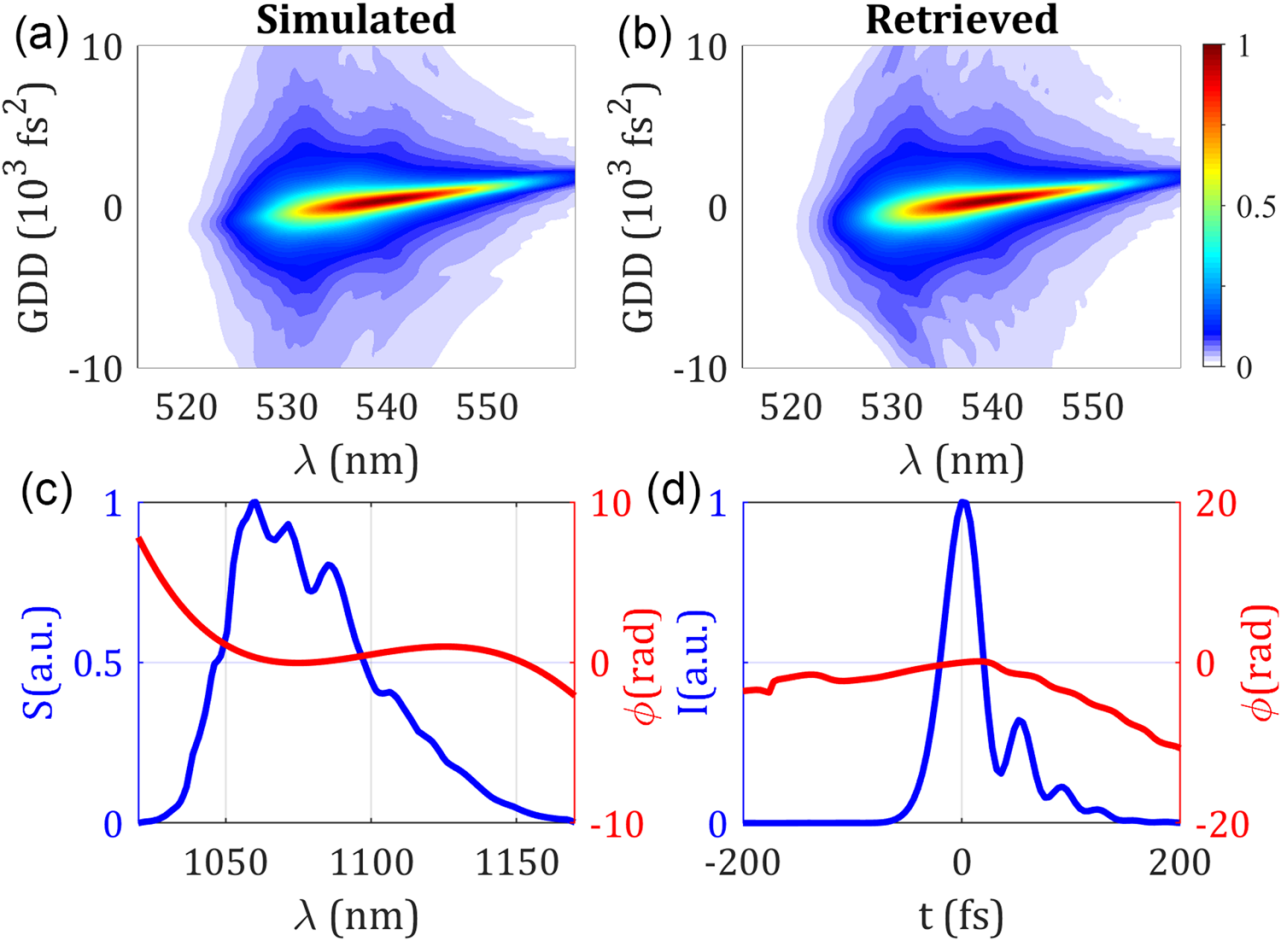
Results for the base pulse. (**a**) Simulated and (**b**) retrieved d-scan traces. (**c**) Simulated spectrum and retrieved spectral phase, and (**d**) retrieved temporal amplitude and phase.

We start by validating the method numerically, using two sets of simulations devoted to two important types of instabilities: group delay dispersion (GDD) and random phase (RND). In the theoretical calculations that follow, we always use the measured spectrum (see Fig. 1c) of the fibre laser used in the experiments presented in the next section for a pump current *I* = 5 A, which gives a bandwidth of 50 nm centred at 1064 nm and a Fourier-limited duration *τ*_0_ = 32 fs FWHM (full-width at half maximum). We simulate a base (initial) pulse with a pure third-order dispersion (TOD) of −25,000 fs^3^ (duration *τ*_*p*_ = 40 fs FWHM), to which the pulse train instability is then added. We chose a non-flat spectral phase of the base pulse to study the effect of the pulse train instability on the retrieved pulse duration compared to the base pulse duration. As a GDD in the pulse would simply shift the d-scan trace in the dispersion axis, we introduced a moderate TOD leading to a 25% increase of the pulse duration FWHM. The known dispersion range of the simulated compressor, *GDD*_*K*_, is 20,000 fs^2^. In all the theoretical results, the retrieved pulse is calculated at an imparted *GDD* = 0 fs^2^. In Fig. 1a we show the simulated d-scan trace of the stable pulse train (for comparison purposes), together with the corresponding retrieved SC d-scan trace (Fig. 1b). Notice that the known simulated spectral phase (Fig. 1c) and the temporal intensity and phase (Fig. 1d) are not shown in the figure as they are equal to the retrieved ones.

**Figure 2:**
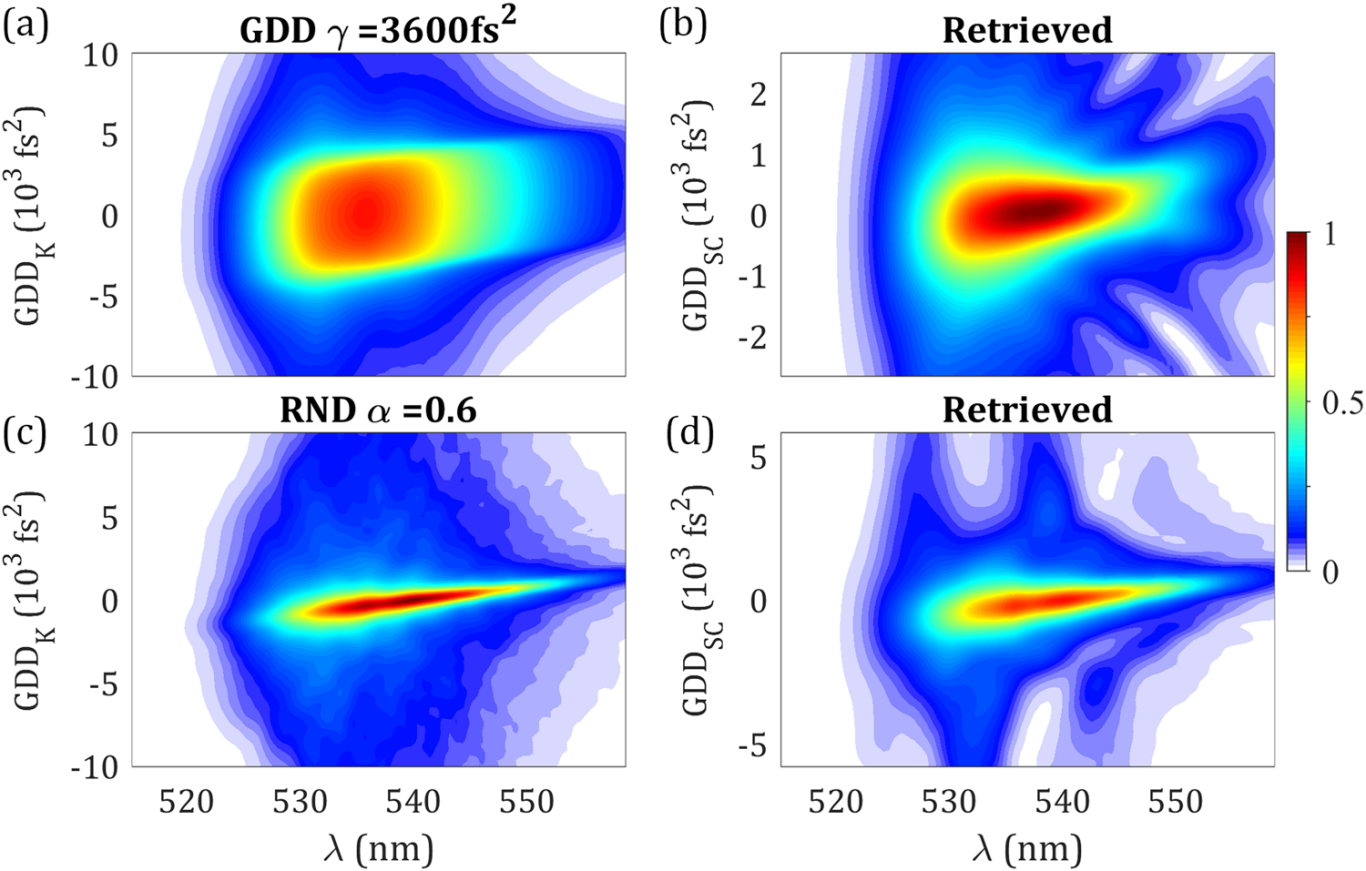
Simulated and retrieved traces of unstable pulse trains. (**a,c**) Simulated and (**b,d**) retrieved d-scan traces, (**a,b**; Supplementary Video 1) for random GDD with $\gamma =3600\,f{s}^{2}$, and (**c,d;** Supplementary Video 2) for random phase (RND) with random instability parameter $\alpha =0.6$.

In a first set of simulations, a GDD instability with magnitude denoted by $\gamma (f{s}^{2})$ is added to the base pulse, with values ranging from 0 to 9000 fs^2^ (for *γ* = 0 we recover the base pulse), where the upper GDD value leads to high instabilities in the pulse train. For each value of *γ*, we simulate a train of 101 pulses with added random values of GDD normally distributed between $\pm \gamma (f{s}^{2})$. We then calculate and average the corresponding d-scan traces. For increasing values of *γ*, the d-scan trace becomes increasingly stretched over the *z*-axis, as seen, e.g., in Fig. 2a (Supplementary Video 1). To estimate the mean pulse duration, *τ*_*γ*_, we calculate it as the temporal duration of the average of the pulse train (i.e., as the average duration of the 101 pulse temporal intensities).

**Figure 3:**
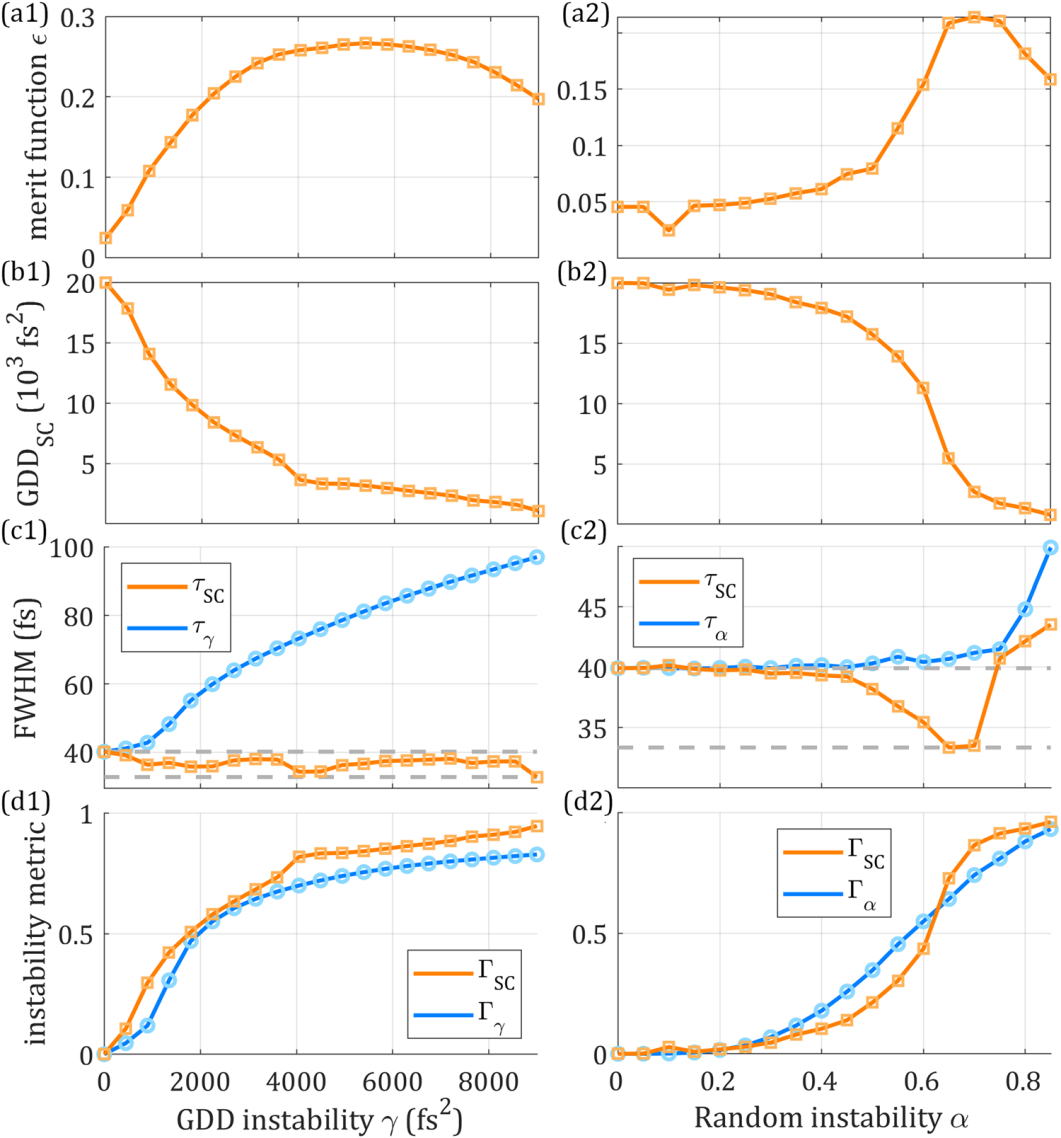
Theoretical results of analysis of the pulse train instability. Due to a GDD instability of varying magnitude *γ* (1^st^ column) and due to a random phase instability of varying magnitude *α* (2^nd^ column). (**a**) Merit function, (**b**) retrieved dispersion *GDD*_*SC*_, (**c**) mean pulse train duration *τ*_*γ*_/*τ*_*α*_ and retrieved pulse duration *τ*_*SC*_, (**d**) instability metrics. In (**b**), the lower and upper dashed grey lines are, respectively, the Fourier-limit duration *τ*_0_ and the base pulse duration *τ*_*P*_.

Despite *GDD*_*K*_ being known, we can apply the SC d-scan algorithm to reconstruct the trace, hence obtaining the SC value for the total dispersion introduced by the compressor, *GDD*_*SC*_. For example, with $\gamma =3600\,f{s}^{2}$, the retrieved trace (Fig. 2b) is clearly stretched compared to the trace in the absence of instability (Fig. 1a), giving $GD{D}_{SC} < GD{D}_{K}$. Notice the different scales in the dispersion axes that reveal this behaviour. This means that the presence of an instability can be directly inferred from the discrepancy between the two GDD values ($GD{D}_{SC}$ and $GD{D}_{K}$). In Fig. 2a,b (Supplementary Video 1), we show the simulated and retrieved traces for different values of *γ*. The complete analysis of these simulations is given in Fig. 3 (1^st^ column). In the absence of instability, the two GDD values are equal. As the instability increases, the ratio $GD{D}_{SC}/GD{D}_{K}$ decreases (Fig. 3(b1)). The retrieved pulse duration, ${\tau }_{SC}$, is always below the base pulse duration ${\tau }_{P}$ (Fig. 3(c1)). The merit function provided by the d-scan error^38^, which we will refer to as *ε*, initially increases with *γ*, but for high instability this tendency is reversed (Fig. 3(a1)), hindering its applicability to the evaluation of the amount of instability. It should be noticed that, both in the simulations and the experiments, for a trace generated by an instable pulse train, there is no retrieved trace that perfectly matches the structure. The retrieved trace corresponds to the *GDD*_*SC*_ accounting for the instability and the described retrieved pulse (shorter than the base pulse).

To quantify the GDD instability in the simulations, we define a quantity ${\Gamma }_{\gamma }=1-{({\tau }_{P}/{\tau }_{\gamma })}^{2}$, which increases with *γ*. We now define the following general metric, which can be obtained from the SC d-scan retrieval provided the introduced dispersion is also known

1 $${\Gamma }_{SC}=1-\frac{GD{D}_{SC}}{GD{D}_{K}}.$$

In Fig. 3(d1), we show the evolution of ${\Gamma }_{SC}$ and ${\Gamma }_{\gamma }$, where both metrics provide similar quantifications of the instability. Also, the new metric ${\Gamma }_{SC}$ is a monotonically increasing function of *γ* (contrarily to the merit function *ε*) further validating the use of ${\Gamma }_{SC}$ as a measurement of the instability.

To cross check these conclusions, we performed a second set of simulations with a different type of instability. To the same base pulse, we now added a normally distributed random phase ${\phi }_{\alpha }({\omega }_{j})=\alpha \cdot rnd(-\pi ,+\pi )$, $\forall {\omega }_{j}$, graded by the random instability parameter, *α*, from 0 to 0.85 (*α* = 0 recovers the base pulse). Here, we also simulated the average d-scan trace of a train with 101 pulses, as shown in Fig. 2c (Supplementary Video 2) for *α* = 0.6, and used the average temporal intensity to calculate the mean duration *τ*_*α*_.

Like in the GDD instability case, the retrieved d-scan trace is stretched for increasing instability and the corresponding retrieval (Fig. 2d) yields $GD{D}_{SC} < GD{D}_{K}$. In Fig. 2c,d (Supplementary Video 2), we show the simulated and retrieved traces for different values of *α*. The complete simulation results of the SC retrievals are given in Fig. 3 (2^nd^ column). In this case, the merit function is also increasing with the instability degree *α* (Fig. 3(a2)), but for high values of *α* it decreases, similarly to Fig. 3(a1). Regarding the retrieved pulse duration, ${\tau }_{\alpha }$, it decreases from the base pulse duration ${\tau }_{P}$ to the Fourier-limit ${\tau }_{0}$, except for high amounts of instability (Fig. 3(c2)). We again find that the retrieved dispersion, $GD{D}_{SC}$, decreases with *α* (Fig. 3(b2)), confirming that it is a good indicator of the degree of instability. In this example, we evaluate the degree of instability as ${\Gamma }_{\alpha }={(1-{I}_{\alpha }/{I}_{0})}^{2}$, with ${I}_{\alpha }$ the peak intensity of the average pulse. Therefore ${\Gamma }_{\alpha }$ is expected to increase from 0 to 1 as $\alpha$ increases. Here, the behaviour of the general metric, ${\Gamma }_{SC}$, is also monotonically increasing and matches the tendency of our definition of degree of instability, ${\Gamma }_{\alpha }$, as shown in (Fig. 3(d2)).

### Experimental application to unstable fibre laser pulse trains

**Figure 4:**
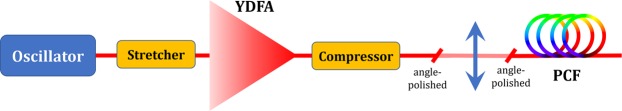
Layout of the fibre laser system. The mode-locked fibre oscillator seeds the chirped pulsed amplification Yb-doped fibre amplifier (YDFA) stage. The YDFA has an angle-polished end to avoid back reflections into the fibre. The output of the amplifier is free-space coupled to a photonic crystal fibre (PCF), where the supercontinuum is generated.

Using the framework established above, we applied SC d-scan to study experimentally the pulse train instability in two different configurations of a broadband mode-locked oscillator-amplifier fibre laser system (Fig. 4). The oscillator is a laser diode-pumped mode-locked fibre laser with a pulse repetition rate of 75 MHz, central wavelength of 1060 nm and spectral bandwidth of 13.6 nm (FWHM). At the output fibre from the oscillator the peak power is P_p_ = 86 W and the temporal width is 3.1 ps (FWHM). In order to avoid nonlinear effects in the subsequent pulse amplification process, the pulses are temporally stretched by means of an optical fibre with normal group delay dispersion (GDD > 0) before the amplifier. The amplifier is an Yb-doped fibre amplifier (YDFA) also with positive GDD, in a fibre-based CPA (chirped pulsed amplification) architecture. After the amplifier the pulses are compressed using a hollow-core photonic bandgap microstructured fibre with anomalous group delay dispersion (GDD < 0), which compensates for the dispersion introduced at the stretching and amplifying stages. The optical properties of the pulsed signal at the end of the compressing stage fiber are the following: spectral bandwidth of 14.5 nm (FWHM), temporal pulse duration of 200 fs (FWHM), average power of 0.3 W, and peak power of 20 kW. For additional spectral broadening, these pulses are free space coupled into a photonic crystal fibre (PCF) at a peak intensity of 45 GW/cm2. The shape and coherence properties of the resulting supercontinuum spectrum show a very strong dependence on the dispersion and nonlinearity characteristics of the PCF, as shown further below.

**Figure 5:**
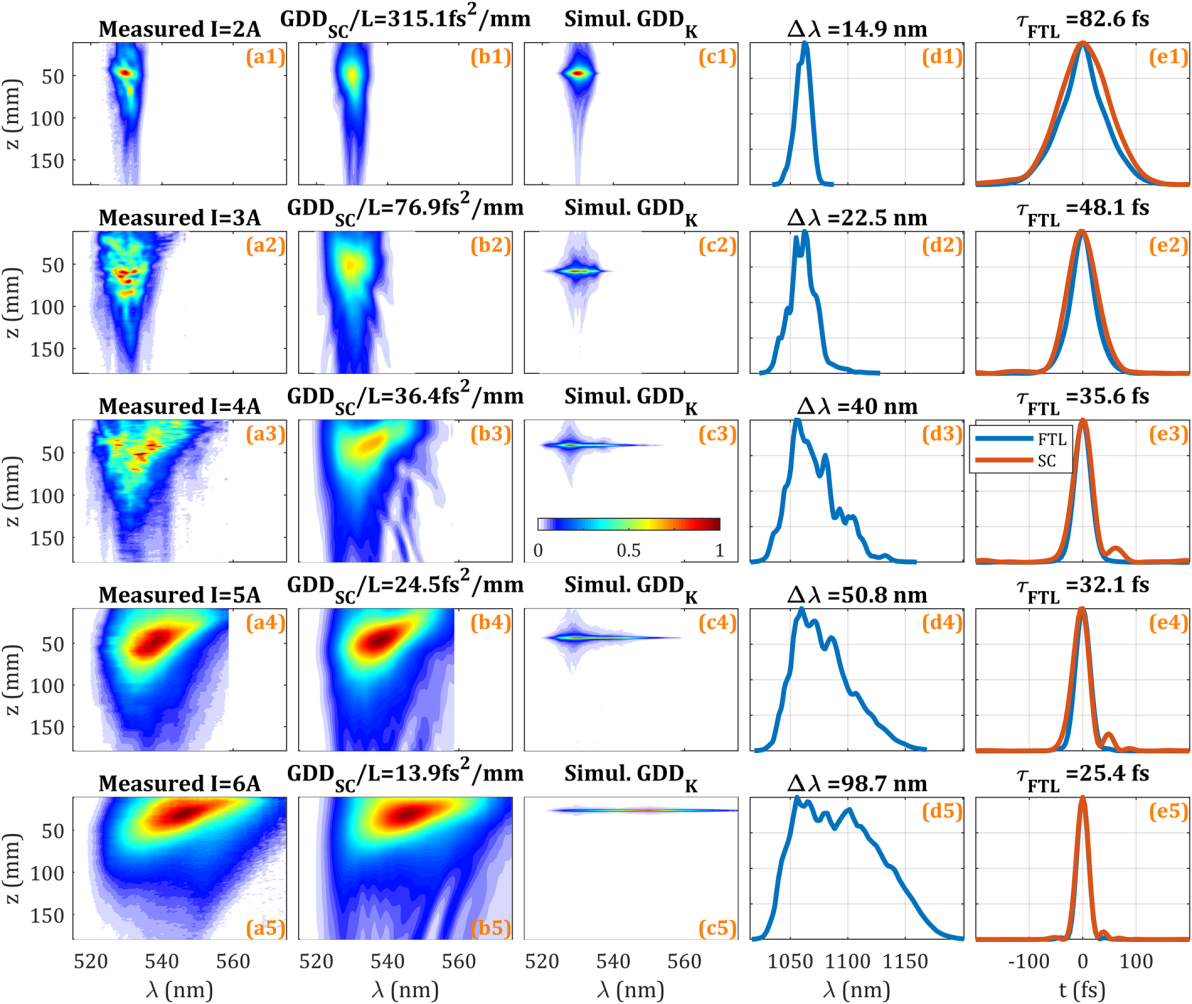
Experimental results for an unstable fibre laser pulse train. (column a) Experimental, (column b) SC d-scan retrieval, (column c) calculated d-scan trace for the experimental spectrum (column d) of the fibre laser at the corresponding pump current assuming a flat spectral phase of the pulse. (Column e) Fourier-limited (FTL) pulse intensity (blue) and SC d-scan retrieved pulse intensity (red). The pump current increases from 2A to 6A from top to bottom row. Note that all traces are individually normalized. Colour scale inset.

In the first configuration of the laser system, the spectral broadening stage used a negative dispersion PCF. We measured d-scan traces for different pump laser currents, from I = 2 to 6A (Fig. 5, rows). The pulses were sent to a grating compressor composed of a 600 lines/mm grating in a four-pass configuration. The inter-grating distance, *z*, was varied over a total range of 170 mm, which provided the amount of dispersion required by the d-scan measurements (Fig. 5, column a). The experimental nominal dispersion (known) introduced by the compressor is $GD{D}_{K}/L=1550f{s}^{2}/mm$, calculated from the geometry of the compressor and the grating groove density^39^. We found that the trace was stretched along the *z*-axis (Fig. 5, column a) when compared to the trace of a stable Fourier-limited pulse train (Fig. 5, column c), which is a clear indication of pulse train instability. When using SC d-scan to retrieve the trace (Fig. 5, column b), we obtained compressor dispersions $GD{D}_{SC}/L$ monotonically varying from 315 to 14 fs^2^/mm as the pump current increases (see the values in Table 1), as expected for increasing pulse train instability.

**Table 1:** Parameters of the unstable pulse train from the fibre laser.

*I*(A)	*GDD*_*SC*_/L (fs^2^/mm)	$\frac{{\boldsymbol{GD}}{{\boldsymbol{D}}}_{{\boldsymbol{K}}}}{{\boldsymbol{GD}}{{\boldsymbol{D}}}_{{\boldsymbol{SC}}}}$	${{\boldsymbol{\Gamma }}}_{{\boldsymbol{SC}}}$	${\boldsymbol{\Delta }}{\boldsymbol{\lambda }}\,({\bf{n}}{\bf{m}})$	${{\boldsymbol{\tau }}}_{{\boldsymbol{FTL}}}$ (fs)	${{\boldsymbol{\tau }}}_{{\boldsymbol{SC}}}$ (fs)
2	315.1	4.9	0.797	14.9	82.6	108.6
3	76.9	20.2	0.950	22.5	48.1	65.6
4	36.4	42.5	0.977	40.0	35.6	40.8
5	24.5	63.2	0.984	50.8	32.1	36.1
6	13.9	111.3	0.991	98.7	25.4	26.5

Despite the similarities (especially for higher pump currents and stronger nonlinear spectral broadening) between measured and SC d-scan retrieved traces (although the algorithm convergence is worse than for traces of stable pulse trains), the dispersion retrieved by the SC d-scan algorithm, $GD{D}_{SC}/L$, was much smaller (from 5 × to 111 × less) than the known dispersion introduced by the actual compressor (Table 1). The large difference in stretching in the dispersion axis scale of the simulated stable Fourier-limit pulse (Fig. 5, column c) compared to the experimental traces (Fig. 5, column a) –e.g., >100 × stretching for *I* = 6 A– is indicative of a high instability of the laser source and provides important quantitative information for its design and optimization. Following our general metric of Eq. (1), we find values of ${\Gamma }_{SC}$ from 0.797 to 0.991, hence confirming the high instability of the pulse train.

These experimental results are complemented by the data given in Table 1. As we increased the pump current, the laser spectrum experienced spectral broadening, $\Delta \lambda$, from 15 nm to 100 nm (FWHM), as shown in Fig. 5 (column d). Therefore, as the Fourier-limit of the pulse, ${\tau }_{FTL}$, decreases from 83 fs to 25 fs, the corresponding trace should be narrower in the z-axis, contrarily to what actually occurs due to the increasing instability. The Fourier-limited pulse and the SC d-scan retrieved pulse are shown in Fig. 5 (column e). The SC d-scan retrieved pulse duration of the unstable pulse train, ${\tau }_{SC}$, is closer to the Fourier limit as the instability increases (see Table 1), being consistent with the numerical simulations. It is remarkable that despite having a varying pulse spectrum (common in nonlinear instabilities), the metric ${\Gamma }_{SC}$ stands correctly for the instability, reinforcing it as a good parameter to evaluate the pulse train instability.

### Optimization of the fibre laser source

**Figure 6:**
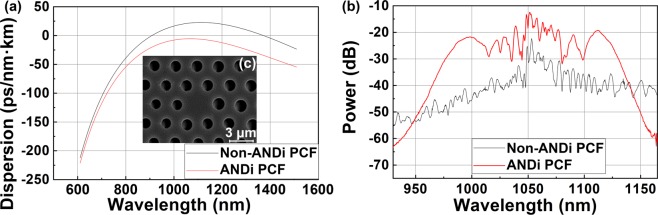
Spectral response and performance of different types of PCF. (**a**) Dispersion curves of the ANDi (red) and non-ANDi (black) PCFs, defined as the variation of group delay with respect to the wavelength per unit length. (**b**) Corresponding spectra (logarithmic scale) of the supercontinuum emission obtained with both types of PCF. (**c**) SEM image showing the general geometry of the PCF.

The pulse train instabilities measured with SC d-scan were identified as originating from nonlinear dynamics within the anomalous dispersion PCF, whereas the generation of stable pulses in fibres is usually achieved in all-normal dispersion (ANDi) schemes^10,17–19,40^. The overall dispersion curve of an ANDi PCF is negative but relatively close to zero over the whole bandwidth (Fig. 6a, red). The dominant nonlinear effect generated under these circumstances is self-phase modulation (SPM), and the spectrum can be broadened without incurring into pulse train instabilities (Fig. 6b, red). On the other hand, when using a PCF with anomalous dispersion (Fig. 6a, black), spectral broadening results from a mix of nonlinear effects, including SPM, stimulated Raman scattering, nth-order soliton breaking, and dispersive wave generation. In these circumstances, the pulsed emission loses its temporal coherence and presents a noisy spectrum (Fig. 6b, black). We should point out that slight differences in geometry of the PCF (Fig. 6c), namely in hole diameter and pitch, translate into marked differences in the spectral broadening behaviour. The required control of the PCF geometry when working close to zero dispersion is indeed at the frontier of current fibre manufacturing technology, which reinforces the need of techniques for determining the level of coherence of the pulses after nonlinear propagation in the PCF.

**Figure 7:**
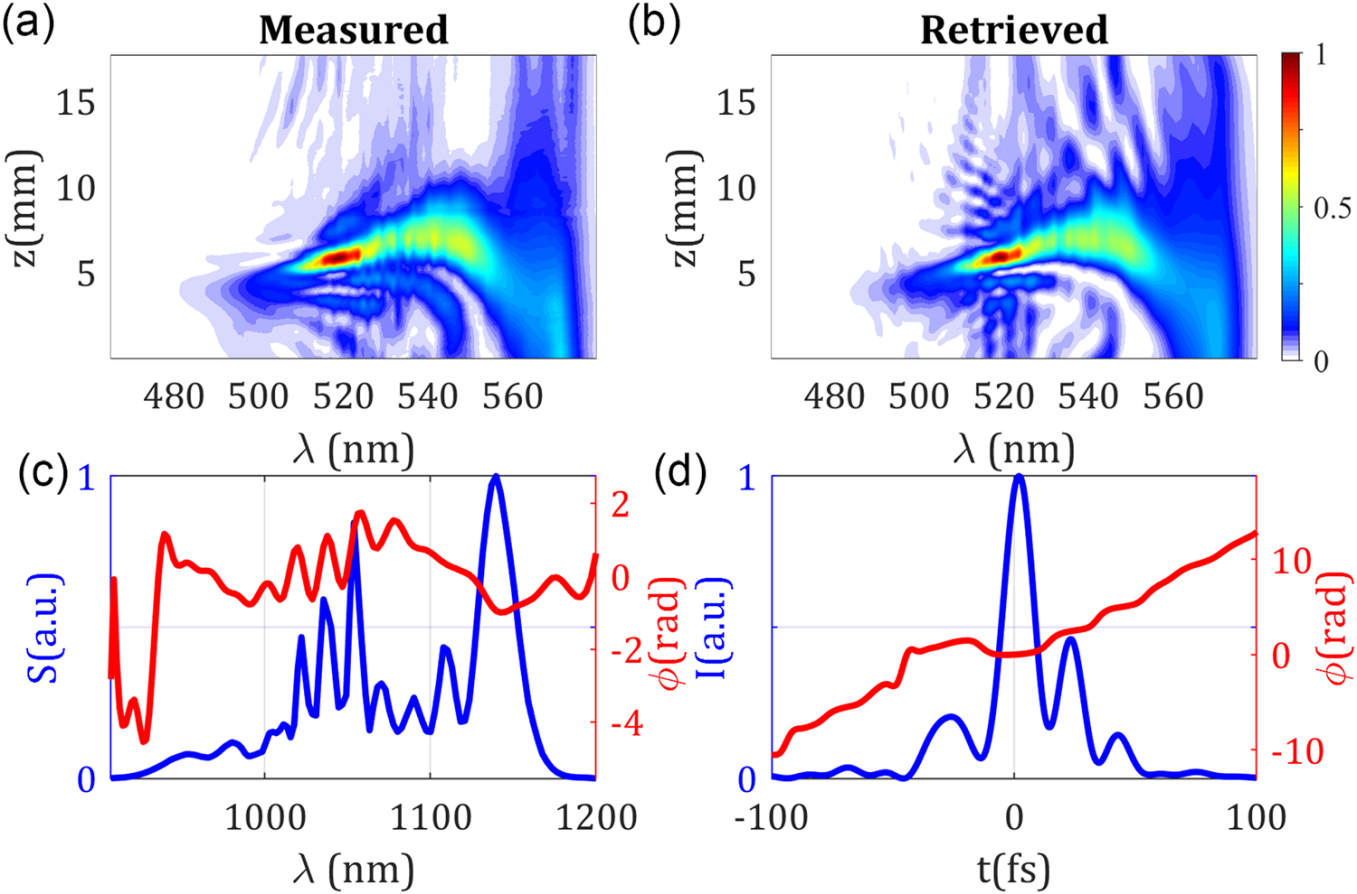
Results for the experimental stable compressed pulse obtained for the ANDi PCF spectral broadening stage. (**a**) Experimental and (**b**) retrieved d-scan traces. (**c**) Experimental spectrum and retrieved spectral phase, and (**d**) retrieved temporal amplitude and phase. The measured pulse duration is 14.7 fs FWHM.

Based on the above information and measurements, we opted for the ANDi PCF as the best choice for an optimized system. In this second configuration, the ANDi regime resulted in broadband spectra with a Fourier-limit of 14.3 fs FWHM (note that the spectral bandwidth is considerably larger than in the unstable cases previously presented). Since the previously used grating compressor would introduce a huge dispersion for these stable pulses, we needed to use a different compressor consisting of a pair of glass wedges and chirped mirrors to perform the d-scan. In Fig. 7 we show the corresponding d-scan results, where the pulse is shown to be well compressed, presenting a relatively small remaining TOD and fourth-order dispersion, as shown by the tilt and the curvature in the trace, respectively. At the optimum compression insertion (5.6 mm), the retrieved pulse has a duration of 14.7 fs (FWHM). Also, the compressor dispersion retrieved with SC d-scan was $GD{D}_{SC}/L$ = 160 fs^2^/mm, which is close to the nominal value of 140 fs^2^/mm estimated from the material and geometry of the wedges, thus confirming the stability of the fibre laser source. As the PCF is seeded with pulses with a Fourier-limit duration of ~90 fs (FWHM), our results show a compression factor of 6. Compared to previous works using PCFs, Hooper *et al*.^18^ measured 26 fs with autocorrelation (14 fs Fourier-limit), or Heidt *et al*.^17^ measured 5 fs with SPIDER (note that pulse train instabilities cannot be discarded in these cases). Other authors did not measure the temporal evolution of the supercontinuum generation^16^.

## Conclusions

In conclusion, we have experimentally shown the capability of self-calibrating (SC) d-scan to evaluate the presence and degree of pulse train instabilities in post-compressed ultrafast fibre lasers. We have identified the origin of the instabilities to be the nonlinear dynamics associated to the anomalous dispersion regime in the PCF used for spectral broadening. Such instabilities can be assessed with a general metric, ${\Gamma }_{SC}$, which is a function of the ratio between the actual introduced dispersion and the SC d-scan retrieved dispersion, and even a simple visual inspection of the measured trace can already reveal the presence of instabilities. This method has enabled us to detect and solve instability issues in a broadband fibre laser by using all-normal dispersion fibres, where we obtained a properly compressed 15 fs stable pulse train. The use of SC d-scan enables integrating the instability detection within the temporal diagnostic, which is very helpful, e.g., for the design and optimization of broadband mode-locked fibre lasers and can also be applied to other laser sources.

The d-scan traces have been shown to be sensitive to different sources of amplitude and phase instabilities both theoretically and experimentally in the present and in previous works^31,32,35,36^. Therefore, one would expect that other sources of instabilities can also be quantified with SC d-scan, for example in solitonic mode-locked fibre lasers^41,42^ or saturable absorber based fibre mode-locked lasers^12,14,43^.

## Methods

### Self-calibrating d-scan technique

**Figure 8:**
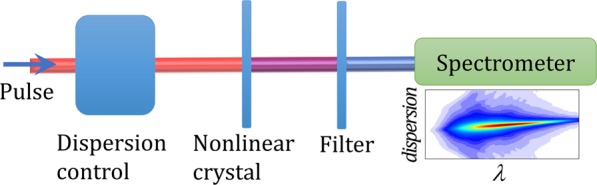
Schematic of the dispersion scan technique. A dispersion module (e.g. a pulse compressor) is used to apply a range of dispersions to the pulse to be measured before generating a nonlinear process (e.g. second-harmonic generation). The filtered nonlinear signal (to remove the remaining fundamental signal) is measured with a spectrometer as a function of the imparted dispersion, resulting in a two-dimensional d-scan trace.

The d-scan technique^33^ can simultaneously compress and characterize ultrashort laser pulses. The electric field of the input pulse to be measured can be written as $E(\omega )=A(\omega )\exp [i\phi (\omega )]$, where its amplitude can be obtained from the measured spectrum, $S(\omega )$, as $A(\omega )=\sqrt{S(\omega )}$, and the spectral phase $\phi (\omega )$ is retrieved from the measurement. In d-scan, a range of dispersions is applied to the input pulse while measuring the spectrum of the second-harmonic generation (or other nonlinear signal) from the resulting pulse (Fig. 8). The dispersion is typically imparted by a pulse compressor, for example a combination of chirped mirrors and glass-wedges^33,44,45^, grating compressors^46^ or prism compressors^37^.

The measured nonlinear signal is a two-dimensional trace, the d-scan trace, being a function of the second-harmonic wavelength (or equivalently the frequency *ω*) and the imparted dispersion. The scanned dispersion ranges from negative to positive and, for an arbitrary spectral phase of the pulse, leads to compression of different parts of the spectrum at different added dispersions. Around optimum pulse compression, the nonlinear signal is higher, while that signal decreases for higher imparted dispersions. The particular structure of the d-scan trace encodes the spectral phase $\phi (\omega )$ of the pulse. The d-scan trace is given by the expression

2 $${S}_{d\cdot scan}(\omega ,z)={| {\mathcal F} [{({ {\mathcal F} }^{-1}\{A(\omega )\exp [i\phi (\omega )]\exp [i\psi (\omega )\cdot z]\})}^{2}]|}^{2},$$

where $\psi (\omega )\cdot z$ represents the imparted dispersion and is parametrized by *z*. Depending on the type of dispersion control, the variable *z* can account for the amount of glass wedge insertion in a wedge compressor, or the variation in distance between dispersive elements in a prism or grating compressor, among others. In SC d-scan^37^, both the pulse phase and the compressor nominal dispersion are simultaneously retrieved. The compressor dispersion per unit length can be expanded in a Taylor series

3 $$\psi (\omega )={\psi }_{0}+{\psi }_{1}\cdot (\omega -{\omega }_{0})+\frac{GD{D}_{tot}}{2L}{(\omega -{\omega }_{0})}^{2}+\frac{TO{D}_{tot}}{6L}{(\omega -{\omega }_{0})}^{3}+\ldots ,$$

where *L* is the total scan range in the variable *z*, with $GD{D}_{tot}$ and $TO{D}_{tot}$ denoting, respectively, the total GDD and TOD introduced during a whole scan by varying the parameter *z* over an amount *L*. The ${\psi }_{0},{\psi }_{1}$ terms, corresponding to the carrier envelope phase and to a net group delay (i.e., a pulse arrival time), respectively, can be ignored as the trace is not sensitive to them. In most cases it is enough to use the GDD and TOD parameters in the expansion given in Eq. (3)^37^. For the SC d-scan retrievals we use the multi-variable optimization Levenberg-Marquardt algorithm (as previously used for SC d-scan retrievals^34,37,47^). We parametrize the unknown phase function, $\phi (\omega )$, in 32 discrete points (interpolated over the complete frequency grid for the calculations), while we model the compressor with pure GDD (a single parameter).

## Supplementary information

Supplementary video 1.

Supplementary video 2.

## Data Availability

The datasets generated and/or analysed during the current study are available from the corresponding author on reasonable request.
